# A Multiple Reaction Modelling Framework for Microbial Electrochemical Technologies

**DOI:** 10.3390/ijms18010086

**Published:** 2017-01-04

**Authors:** Tolutola Oyetunde, Priyangshu M. Sarma, Farrukh Ahmad, Jorge Rodríguez

**Affiliations:** 1 Department of Chemical and Environmental Engineering (CEE) Masdar Institute of Science & Technology, P.O. Box 54224, Abu Dhabi, United Arab Emirates; tolutoyo@gmail.com (T.O.); fahmad@masdar.ac.ae (F.A.); 2 The Energy and Resources Institute (TERI), Darbari Seth Block, India Habitat Centre, New Delhi 110 003, India; priyangshumsarma@gmail.com

**Keywords:** bioelectrochemistry, modeling, bio-electrosynthesis, bioremediation, resource recovery

## Abstract

A mathematical model for the theoretical evaluation of microbial electrochemical technologies (METs) is presented that incorporates a detailed physico-chemical framework, includes multiple reactions (both at the electrodes and in the bulk phase) and involves a variety of microbial functional groups. The model is applied to two theoretical case studies: (i) A microbial electrolysis cell (MEC) for continuous anodic volatile fatty acids (VFA) oxidation and cathodic VFA reduction to alcohols, for which the theoretical system response to changes in applied voltage and VFA feed ratio (anode-to-cathode) as well as membrane type are investigated. This case involves multiple parallel electrode reactions in both anode and cathode compartments; (ii) A microbial fuel cell (MFC) for cathodic perchlorate reduction, in which the theoretical impact of feed flow rates and concentrations on the overall system performance are investigated. This case involves multiple electrode reactions in series in the cathode compartment. The model structure captures interactions between important system variables based on first principles and provides a platform for the dynamic description of METs involving electrode reactions both in parallel and in series and in both MFC and MEC configurations. Such a theoretical modelling approach, largely based on first principles, appears promising in the development and testing of MET control and optimization strategies.

## 1. Introduction

Environmental biotechnology relies largely on the use of mathematical models for process design, operation, optimization, and control [1]. For successful microbial electrochemical technologies (METs) scale-up and deployment, a better understanding of the system and potential bottlenecks in the operation are required. Mathematical models can help uncover nontrivial interactions between the biological, electrochemical, and physical processes involved [2].

The level of detail of any model is determined by the specified modeling objectives. MET modeling objectives can vary widely but generally include the relationship between possible manipulatable variables (e.g., influent concentrations, flows, pH temperature, external resistance) and variables that define system performance (e.g., current density, power density, and reaction rates). In contrast to the objectives of “models for prediction/forecasting”, “models for understanding” [1] would seek to shed more light on the basic bioelectrochemical phenomena in METs. Overall, MET modeling has been receiving attention and several MET models have been published in literature over the past decade [2,3,4,5,6,7,8].

Recently the importance of modeling for the development of METs has been highlighted and how very few models exist compared to experiments [9]. MET models are valuable tools for detailed studies and they also provide a platform for systematic integration of knowledge gained by experimentation.

Specialized models are also being developed for different types of MET [10,11,12,13]. Recent efforts are focusing on model-based control system design and optimization of MET [14,15]. This is important because dynamic control strategies have been shown to improve MFC efficiency and minimize losses [16].

The aims of most of previously published models have been to:

1. Predict kinetics in terms of current and voltage of the system as functions of operation conditions.
2. Describe and study the mechanism of extracellular electron transport: conductive biofilms, nanowires, electric gradient, electron shuttling, etc.
3. Investigate the ecology of the microbial communities that perform extracellular electron transport (EET).

These models have achieved different levels of utility within their range of applicability. There are, however, important limitations, including models very specific to reactor configurations and conditions, large number of parameters (many of which have no mechanistic interpretation or suffer from identifiability problems), long simulation times, or gross oversimplifications. MET modeling requires structures that are adaptable (due to the ever-increasing knowledge of bioelectrochemical phenomena) and fast to implement. Among the published models, few appear to have achieved a good balance between an accurate system description and speed of simulation, especially for multi-population, multi-substrate METs involving multiple electrode reactions either in series or in parallel. A major limitation is the difficulty in predicting electrical current considering the numerous variables involved.

Moreover, there appears to be little evidence to suggest that the present crop of models have uncovered any non-trivial interactions among the different biological, physical, and chemical variables [9,17]. MET modeling has so far failed to reach its full potential due to several challenges, including lack of reproducible and mechanistically meaningful frameworks for parameter identification (especially for the electron transfer mechanisms, microbial ecology, bioenergetics and kinetics) and model validation [3]. Thus, while predictive MET models [5,6,18] provide good agreement with experimental measurements, they are not applicable outside their domain of construction and offer little mechanistic insights. Most of these models do not explicitly capture transport across the membrane, pH changes and changes in the gas phase composition. On the hand, MET models whose main purpose is to uncover nontrivial interactions between biological, chemical, and electrical phenomena contain too many parameters and are usually computationally intensive [2,4,7,8,19]. Thus, limiting their application for scanning different possible configurations of METs for varied applications. This is the gap that we intend to bridge. Our approach aims to be both mechanistically insightful and useful as a predictive tool. We achieve this by capturing the partition of electrons (in the form of current) from substrates to products based on mass balance principles. This allows us to account for chemical changes (including changes in pH) as well as model substrate kinetics in a multi-species, multi-phase framework. Furthermore, our method defines the individual contribution of different chemical and biological species to current production and consumption, which has not been reported to date.

With renewed interest in prospective microbial electrosynthesis processes for biofuels/chemical production from diverse substrates, there is a need for models capable of accurately describing the chemistry of multiple reactions at the electrodes and using the current as input instead of focusing on its prediction. This would enable the evaluation of prospective METs in terms of their detailed chemical changes, including pH and accumulation of products. Such models would not only help as selection tools for interesting applications but also lend themselves to calibration by well-established electrochemical techniques.

## 2. Results and Discussion

In this section, example model simulation results are presented to illustrate the model’s capability of describing well-characterized behavior of both MFCs and MECs under the two theoretical case studies.

### 2.1. Ethanol/Butanol Production in an MEC

**Figure 1:**
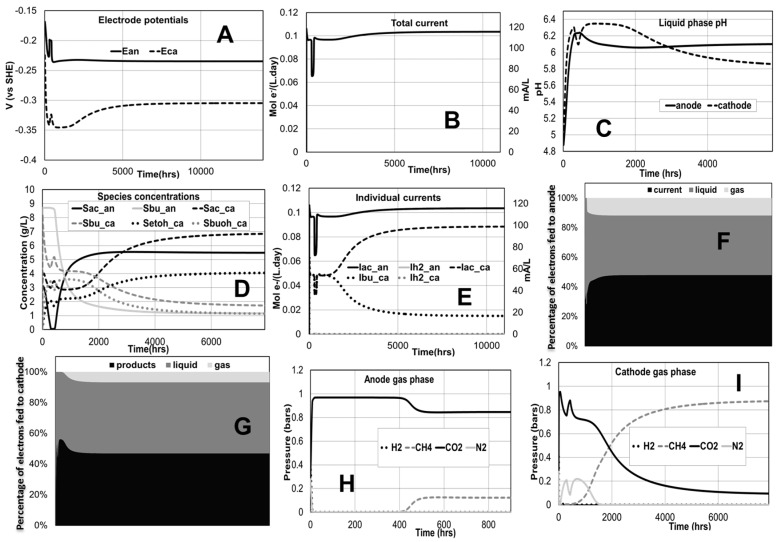
Simulated time evolution of selected variables of interest for the ethanol/butanol base case simulation. (Applied voltage of 0.7 V; total feed flow rate of 0.09 L/day containing 0.1 M of acetic and butyric acids; anode-cathode feed ratio is 1:3). (**A**) Anode and cathode potentials; (**B**) Total current; (**C**) pH in anode and cathode compartments; (**D**) Concentrations of different chemical species. (**E**) Individual current contributions; (**F**) Fate of electrons in the anode; (**G**) Fate of electrons in the cathode; (**H**) Anode gas phase composition; (**I**) Cathode gas phase composition.

**Figure 2:**
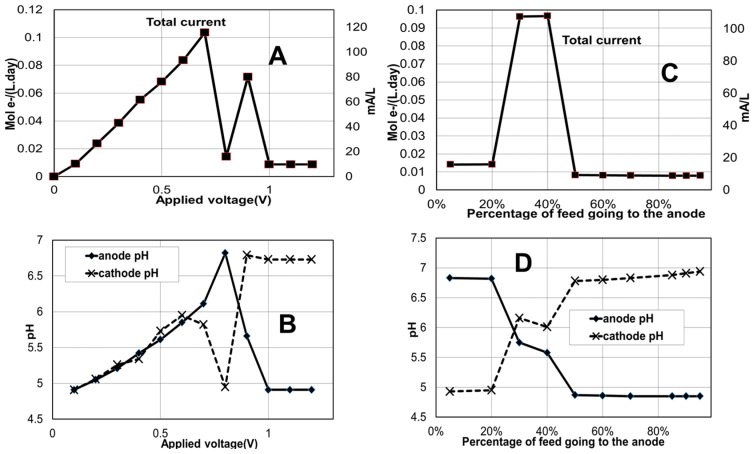
Effect of varying applied voltage (**A**,**B**) and anode-to-cathode feed ratio (**C**,**D**) on total current and pH of the system. (Total feed flow rate of 0.09 L/day containing 0.1 M of acetate and butyrate; proton exchange membrane is used. For graphs (**A**,**B**) anode to cathode feed ratio is 1:3 and for graphs (**C**,**D**) applied voltage is 0.7 V). Each point is the steady-state value that is reached upon imposition of the respective feed profile.

Figure 1 shows the model base case simulation results for the ethanol/butanol MEC case study (with three times as much substrate going to the cathode as to the anode and for an applied voltage of 0.7 V). The anode potential is predicted higher than the cathode potential due to the external power source in this MEC configuration (Figure 1A). The predicted difference between the anode and cathode potential (~0.07 V) is only a fraction of the applied voltage due to the internal and external resistances also accounted for. The model also describes the contribution of the different substrates to the total current (Figure 1B,E). In this specific case hydrogen (and proton ions) is not predicted to have a significant contribution to the current observed. Also in this case, the pH values both at anode and cathode compartments allow for the growth of methanogens (Figure 1C). pH is also predicted higher at the anode than at the cathode because of the relatively smaller inflow (of acidic acetate and butyrate) to the anode. These predictions suggest for example a possible design to simultaneously produce both biogas and liquid fuels if the economics are favorable. The partial pressure of methane in the cathode (Figure 1H,I) is predicted much higher than that in the anode because the feed flow rate to the cathode is three times higher, providing much larger electron donor substrate available for methanogenesis. The high production of methane at the cathode (Figure 1I) is consistent with experimental studies of reduction of volatile fatty acids [20]. Figure 1F,G illustrate the predicted relative fate distribution of the electron equivalents fed top the cathode and the anode compartments into either current (or product) or into liquid and gas phase products. These simulation results illustrate the capacity of the model to describe these complex interactions at a theoretical level and serve as a tool for optimization or control strategies design to drive electrons towards current (in the anode) and products (in the cathode). Interestingly, the current production from acetate is about three times that of butyrate in the cathode (Figure 1E). This agrees well with experimental measurements of ratio of current generation from butyrate to acetate (2.85 in [21]). Moreover, the production of ethanol from acetate at a cathode sustained at −550 mV vs. Ag/AgCl electrode was about 13.5 mM in similar settings to our model [22]. Applying about 0.8 V in our simulation gives a comparative cathode potential (~−435 mV vs. Ag/AgCl) and 15.2 mM of ethanol. Our simulations allow scanning different potentials for production sweet spots (Figure 2).

Butyrate utilization is not predicted in the anode until almost full acetate depletion due to thermodynamic feasibility constraints of the butyrate to acetate fermentation. Electroactive consumption of both acetate and butyrate in the cathode is predicted. In the simulated base case, the product stream is expected as richer in ethanol (about 3.7 times higher concentration than butanol) (Figure 1D), suggesting that the feed ratio optimization can be manipulated with implications in the downstream separation operations and overall economics of the process. This ratio of ethanol to butanol production is comparable to experimental measurements of a similar system [23].

#### 2.1.1. Effect of Applied Voltage

The capacity of the model to describe the effect of the applied voltage on the system performance was evaluated by simulating the process at the base case conditions above but changing the applied voltage from 0.1 to 1.2 V in steps of 0.1 V, in different runs. The steady-state results for each applied voltage are presented in Figure 2A,B. Figure 2A shows how the model predicts overall current increases with increasing applied voltage up to a value in which the current nears the influent flow rate of electron equivalents (as substrates) into the system. An interesting bounce-back effect is described at a later higher voltage 0.9 V attributed to that voltage allegedly helping maintain a lower pH in the cathode (as seen in Figure 2B) inhibiting methanogenic competition for electron equivalents. Thus, pH optimality has a strong influence on power generation and coulombic efficiency. This effect has been experimentally observed in several studies [24,25,26].

#### 2.1.2. Effect of Anode-to-Cathode Feed Ratio

In this case study, only one stream is fed to the system split part into the anode and part into the cathode. Changing the anode-to-cathode feed flow ratio is equivalent to regulating the hydraulic residence times at these compartments and appears as a potentially low-cost control variable for this process. The model is demonstrated in its capacity of describe the impact of this variable on the overall system by simulation at different values of percentage of the total feed to the anode (from 5% to 95%). The steady-state model predictions are presented in Figure 2C,D.

It is important to highlight that very low or very high ratios (10%, 15% and 80% fed to anode) led to unstable model response (data not shown) likely due to the strong substrate limitation imposed in one or the other electrode leading to predicted depletion of both acetate and butyrate at the limited electrode. The model predicts the optimum percentage of feed flow to anode to be somewhere between 25% and 40% (Figure 2C). A relatively high concentration of products as well as higher bioelectroactivity is also expected in this optimum range. Below this range, the pH would become too high in the anode and above this range, the same would occur in the cathode (Figure 2D). The alkaline pH values lead to favored methanogenesis and loss of electrons equivalents away from electrode reactions and into methane.

### 2.2. Perchlorate Remediation in an MFC

**Figure 3:**
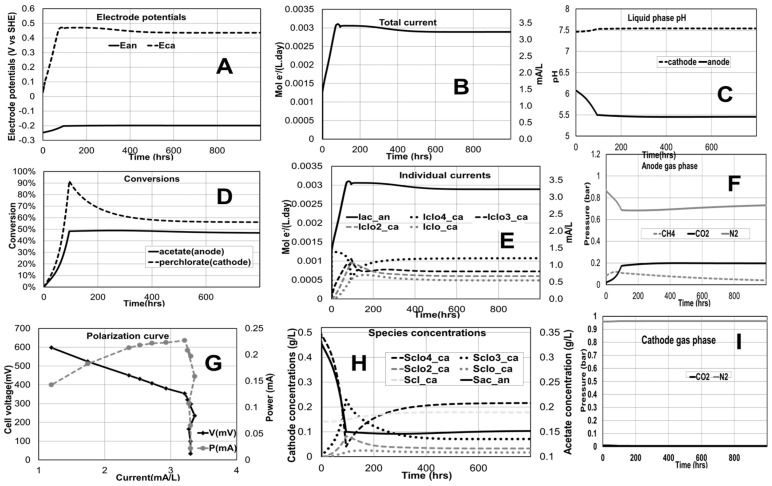
Time evolution of variables of interest for perchlorate remediation base case simulation (Anode: inlet flow rate −300 mL/day, acetate concentration −350 mg/L, volume −200 mL; Cathode: inlet flow rate −300 mL/day, perchlorate concentration −350 mg/L, volume −200 mL; proton exchange membrane used; external resistance is 419.01 ohms). (**A**) Anode and cathode potentials; (**B**) Total current; (**C**) pH in anode and cathode compartments; (**D**) Conversions of chemical species (**E**) Individual current contributions; (**F**) Anode gas phase composition; (**G**) Polarization curve; (**H**) Concentrations of the different chemical species; (**I**) Cathode gas phase composition.

The base case simulation results of the perchlorate remediation MFC are presented in Figure 3. The model describes the time profile of all the important chemical and electrical variables. In this case study, the cathode potential is higher than the anode potential as it is an MFC configuration (Figure 3A). The cathode pH slightly increases with corresponding decrease in perchlorate concentration (increase in removal efficiency). This pH-removal efficiency correlation has been observed experimentally [27]. The modeled individual current contributions (from the four electrode reactions in series) to the total current (Figure 3B) are shown in Figure 3E. The predicted substrate concentrations (Figure 3H) and conversions (Figure 3D), in this specific conditions, show acetate in the anode not fully consumed suggesting that the system could function with a lower inlet acetate concentration and/or flow. The pH predicted in this example for the anode (Figure 3C) would be sufficiently low to prevent the activity of methanogens, and the one in the cathode within 7–9 would be optimum for perchlorate reducing bacteria [28].

Polarization curves are also generated using the model. Steady-state values of the voltage, current and power for different external resistances (between 50 and 2500 ohms) are computed (Figure 3G). The simulated polarization curve shapes are in line with expected experimental polarization plots describing the inverse relationship between current and voltage as well as the existence of a maximum the power generated [29,30].

#### Effect of Influent Flow Rates and Concentrations

**Figure 4:**
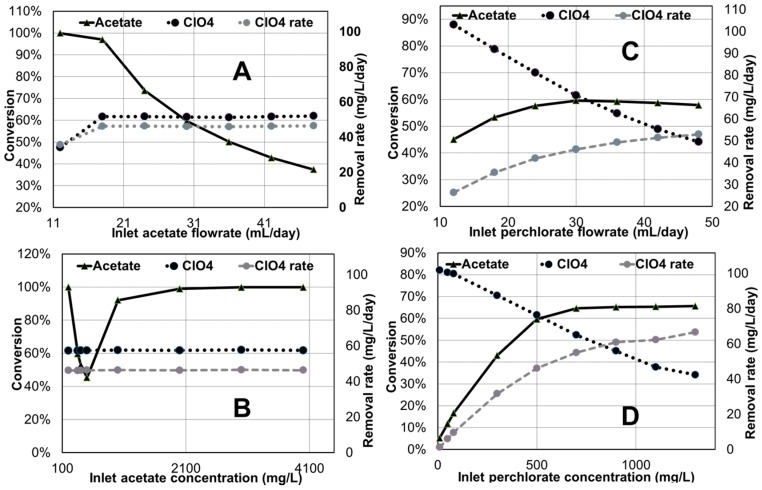
Influence of different variables on conversion for perchlorate remediation base case simulation (Anode: inlet flow rate −300 mL/day, acetate concentration −350 mg/L, volume −200 mL; Cathode: inlet flow rate −300 mL/day, perchlorate concentration −350 mg/L, volume −200 mL; proton exchange membrane used; external resistance is 419.01 ohms). (**A**) Effect of inlet acetate flow rate; (**B**) Effect of inlet perchlorate flowrate; (**C**) Effect of inlet acetate concentration; (**D**) Effect of inlet perchlorate concentration.

Predicted steady-state acetate and perchlorate conversions under different feed flows and concentrations are presented in Figure 4. An increase in expected perchlorate conversion up to a certain value is predicted for increasing anode feed acetate concentration and/or flow rate, beyond which there is no further impact on perchlorate conversion (Figure 4A,B). The model however also describes how higher acetate concentrations can result in higher acetate conversions uncoupled to additional perchlorate conversion due to pH conditions favoring methanogenesis. Higher perchlorate influent flow rates to the cathode are predicted to increase removal rates but at lower perchlorate conversions (Figure 4C). A similar trend for increasing influent perchlorate concentrations is also predicted by the model (Figure 4D). The simulation results shown in Figure 4 suggest that perchlorate conversion could be optimized by configurations in which, for example, acetate and perchlorate rich streams could move in counter current flow through compartments in series or in a tubular fashion. These model-based quantitative and qualitative findings could prove very valuable in designing process configurations and control strategies.

## 3. Materials and Methods

### 3.1. Model Structure Selection

For a complete description of an MET system, several key elements must be modeled including electric circuit (potential losses), bulk hydraulics, biofilm, abiotic electrode reaction kinetics, bio-electrode reaction kinetics, mass transport, ion-selective membrane transport, electron transfer mechanism, microbial activity, metabolism, and ecology [3].

The proposed model structure consists of a continuous-stirred tank reactor for both anode and cathode compartments with space-independent properties in which Nernst-Monod-based equation is used for the electrode kinetics (like what is described in [4,19]). A diffusion layer is assumed around the electrodes with a loss factor model (replacing the more complicated and parameter rich Butler-Volmer equation) for the activation losses. The biofilm is not spatially described as such but only biomass retention (via an imposed solids retention time (SRT)) with a cap maximum concentration of active biomass in the system, above which biomass leaves the system. The model does not focus on the mechanisms by which extracellular electron transport occurs but rather the detailed account of the effects of such transfers on the biological and chemical speciation. The main input variables are temperature, pH, substrate flows and concentrations, hydraulic retention times, external resistance, and applied voltage (for MECs) [18].

The model, based on a system of differential algebraic equations (DAE), describes the dynamics of the state variables in four domains, namely anode and cathode liquid phases, and anode and cathode gas phases. Both anodic and cathodic bulk liquid compartments are assumed as separate perfectly mixed reactors with suspended biomasses with a defined solid retention time to mimic biofilm associated retention. The general form of the DAE is shown in Equations (1)–(3) for the liquid, solid and gas phases respectively. *X* is an “m × 1” vector of the so called “state variables”, chemical and biological concentrations in a domain; *D* is the “dilution rate” (typically equal to the ratio of inlet flow rate to liquid volume). *R* is a “m × 1” vector of generation terms accounting all reactions (both electrode reactions and pure microbial fermentations) and transport processes (e.g., transport between anode and cathode chambers across the membrane, transport between liquid and gas phases) referred to each concentration of the vector *X*. For suspended solid states (Equation (2)), a solid retention time parameter and outflow factor is used to determine the biomass outflow rate. The outflow factor depends on two parameters: the maximum concentration of active biomass, *X_max_* and a half saturation factor, *k*. For gas phase states (Equation (3)), *X* is expressed in units of partial pressure. *P* and *P_w_* are the total and the water vapor pressure respectively. The generation terms *R* is computed as the product of a Petersen-like “m × p” stoichiometry matrix *M* (Petersen, 1965) and the “p × 1” vector of reaction and transport rates *r*. Reaction and transport rates are computed from kinetic and transport rate expressions, which depend on the state variables on so-called “algebraic states” (computed algebraically from the state variables at every instant of time e.g., pH, thermodynamic variables, electrical potentials, etc.)

(1) $$\frac{dX}{dt}=D\times \left(X_{in}-X\right)+R\;,\;R=M.r$$

(2) $$\frac{dX}{dt}=D\times \left(X_{in}-X.\left(\frac{f}{D\times SRT}\right)\right)+M.r\;,\;f=1+\frac{k\times \sum X}{\left(X_{max}-\sum X\right)}$$

(3) $$\frac{dX}{dt}=R_{c}T.\left(\frac{V_{liq}}{V_{gas}}\right)\times \left(M.r-\frac{X\times \sum \left(M.r\right)}{P-P_{w}}\right)$$

The computation of the algebraic state variables at each instant of time is based on the specific modelled processes, for which details are provided in the following sections: (i) acid-base ionic speciation ensuring charge neutrality and chemical equilibria, including pH calculation; (ii) electrical model for multiple electrode reactions at anode and cathode; (iii) selective transport of ionic species between chambers through the separation membrane. All parameters used in the model are described in the Table S1.

### 3.2. Generalized Physico-Chemical Framework for Bioprocess Modeling

To facilitate a rigorous description of the physical and chemical changes of all relevant species in an MET, the model developed uses a chemical ionic speciation solver to compute the concentrations of all the ionic species. This solver is based on a generalized algorithm, utilizing only thermodynamic information of the chemical species to numerically compute the pH and ionic speciation of any aqueous solution. Only standard Gibbs energies of formation of the species are required (along with enthalpies, but only if temperature effect is to be considered). Details of this framework are described in [31,32,33].

### 3.3. Modeling Competing Anaerobic Fermentative Processes

To fully describe the chemical changes occurring in METs, any relevant competing non-electroactive microbial reactions must also be accounted for. In this case, equations from the well-known IWA ADM1 model [34] have been used to describe the non bioelectrochemical anaerobic microbial activities in terms of microbial stoichiometry and kinetics, modified by thermodynamic terms to stop reactions when thermodynamically unfeasible.

### 3.4. Electrode Reaction Kinetics

Biolectrochemical kinetics are described by Monod-like terms for substrate limitations and inhibitions (such as pH). A specific term accounts for the quality of the electrode catalysis through a half-saturation constant KSE for the needed activation overpotential [19], (see Figure S1). The concentrations used for the kinetics are those at the surface of the electrode as algebraically computed from the bulk (state variable) concentrations and their diffusion rates at each time step. This approach allows for the description of the quality of the overall bioelectrochemical catalysis through one single semi-empirical parameter KSE that can be adjusted to match the observed current in the system. The use of KSE to directly match current as input is recommended so that the remaining processes taking place can be accurately described through mass balances and thermodynamic equilibrium, lowering the model’s uncertainty.

### 3.5. Electrical Model (Modeling Multiple Electrode Reactions)

**Figure 5:**
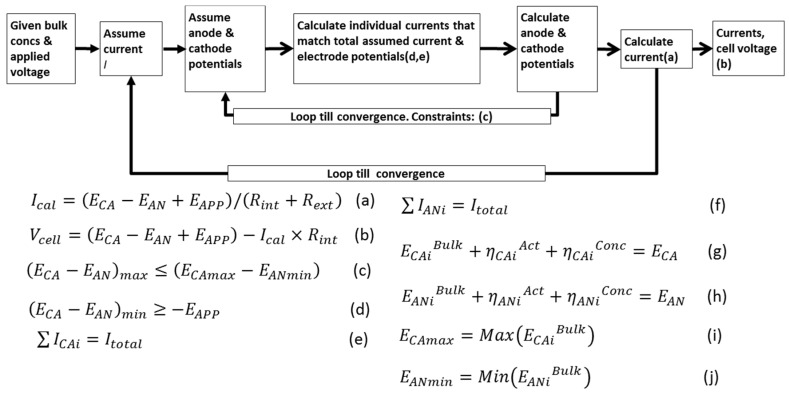
Electrical model structure and solution procedure in the integrated microbial electrochemical technology (MET) system allowing for multiple electrode reactions. More details presented in Figure S2.

The proposed modeling strategy combines a detailed physicochemical speciation framework to account for all chemical and biological species present. A previous effort [35] sought to address this challenge by integrating an ASM2d-activated sludge model and a one substrate MFC model. Our approach goes beyond this by providing a generalized framework for multiple substrates and species for both current-generating (MFC) and current-consuming applications. A strategy is presented to compute current contributions from each reaction such that the observed current matches overall current balance, and the potentials are consistent for all reactions involved at one electrode [36]. A case study is presented using simultaneous cathodic reduction of acetate and butyrate to alcohols and simultaneous anodic oxidations of acetate and hydrogen [23]. The strategy is illustrated in Figure 5. The modeled system was setup such that two fundamental rules are always met:

(i). In each electrode, the individual current contributions of each electroactive reaction must add up to the observed total current; and
(ii). The electrode potential is unique for all reactions taking place at that electrode. This means that the sum of the reaction potential at the bulk plus the potential losses both by concentration polarization and activation for each reaction must match a unique electrode potential both for the anode and for the cathode.

### 3.6. Modeling Competition between Electroactive and Non-Electroactive Microbial Species

Most MET models are based on single electroactive species utilizing a single substrate (usually acetate). This was appropriate because the predominant modeling objective was to predict the magnitude of electric current and voltage due to microbial electroactivity. Some authors have recently proposed a multi-population MET model for one substrate [5,37]. However, in real systems that would have to be designed and controlled, because different microbial species compete for multiple substrates which undergo changes in a metabolic network. This is achieved by defining specific state variables for each microbial group and substrate in the system. The model can therefore describe competition for substrates and between microbial populations as dictated by the chemistry and thermodynamics.

### 3.7. Modeling Ionic Flow across Different Types of Membranes

It is well known that the type and surface area of membranes used in METs have a significant effect on overall performance [38]. This is because the membrane bears a voltage loss depending on the nature and the composition of the ionic medium. The membrane also builds up a pH difference between anode and cathode compartments due to the charge balancing not taking place solely by proton (or hydroxyl ion) diffusion but also by other mobile and abundant ions like sodium and potassium ions present in the medium [39]. Some researchers have attempted to experimentally quantify and qualify relationship between membrane type (and surface area) with cell performance for specific systems [40,41,42,43,44].

Although a detailed modeling of the ion transfer across membranes has already been addressed [38], the long computation times required for the model limited its applicability [38]. In the present work, a simplified ionic flux based on the principle of charge neutrality was used by imposing the condition that the total electrical current equals the total ionic transport through the membrane. The transport numbers are calculated for the different charge carriers based on their relative permeabilities across the membrane as well as their electrical conductivities (function of the concentration and molar conductivity of the species). Detailed equations for this ionic flow across membranes are described in the Figure S3.

## 4. Description of Case Studies

Two theoretical MET case studies are presented to illustrate the applicability of the model for MET, including cases for multiple electrode reactions in parallel and in series.

### 4.1. Ethanol/Butanol Production in an MEC

Anaerobic fermentation has proven to be a relatively successful and low cost process for the treatment of a wide variety of organic waste streams and for the recovery of valuable products (usually biogas) using mixed cultures [45]. Given the diminishing value of methane in today’s energy market, much attention has been focused on the utilization of side streams from anaerobic digestion, which consist primarily of volatile fatty acids (VFAs). The biological conversion of VFAs into more reduced products represents a potential sustainable route for production of valuable liquid fuels from low-value waste streams.

Initial research efforts reported low efficiencies when utilizing hydrogen as an electron donor in the conversion process [20]. The use of a solid electrode as electron donor for this process has shown good promise, though the conversion rates and product concentrations achieved so far remain relatively low, and an electron mediator is usually required [22]. Recent work has, however, shown the possibility of utilizing stable halophilic sulfate-reducing bacteria (SRB)-mixed cultures for higher conversion rates [23]. Key challenges identified with the bioelectrochemical conversion route include the need to limit the diversion of substrates into the many possible undesired reactions as well as a need to ensure low biomass yields [46]. A robust control strategy appears as critical to ensure a bioelectrochemical system with competitive conversions.

In addition to further experimentation, a mechanistic model capable of predicting chemistry and microbial competition rather than focusing simply on current prediction can be utilized as a low uncertainty tool, essential to advancing process development including process configuration selection as well as effective monitoring and control architectures. The modeling approach described aims at addressing these aspects for a technical evaluation of a process that describes the multiple electrode and non-electrode reactions under a rigorous material balance and chemical speciation foundation.

**Figure 6:**
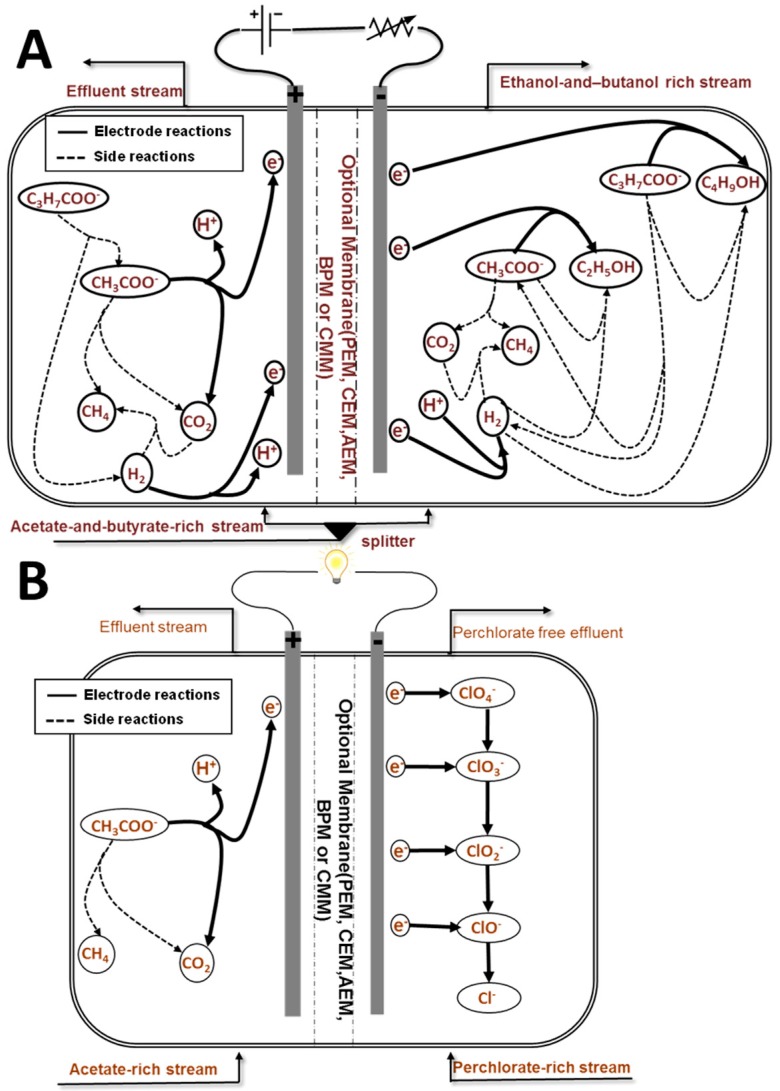
Proposed reactor configurations and reaction network for the (**A**) ethanol/butanol and (**B**) perchlorate reduction case studies. Details of the reactions are shown in Figures S4 and S5.

The data and findings from previous experimental and modeling studies [18,20,21,22,23,24] have been used to tailor the model structure for this specific process. The reaction network considered is presented in Figure 6a. The reactions were selected based on published experimental results as well as thermodynamic considerations. They included the electroactive oxidation of acetate and hydrogen in the anode and the electroactive reduction of acetate, butyrate and protons in the cathode. The reduction of acetate and butyrate by hydrogen in the cathode is also modeled. Hydrogenotrophic and acetoclastic methanogenesis as well as butyrate reduction to acetate are also accounted for in both the anode and cathode. The kinetic parameters for non-electrode microbial reactions were based on ADM1 [34].

### 4.2. Perchlorate Remediation in an MFC

Perchlorate ion poses a growing concern because of its mobility and stability in the environment (ground and surface water) and the negative impact it has on human health if ingested with drinking water. Biological processes for perchlorate remediation have proven to be efficient and economical [47]. The microbial activity in these processes typically uses perchlorate as the terminal electron acceptor coupled with an organic electron donor such as acetate [48]. The use of a microbial fuel cell provides the distinct advantage of decoupling the acetate oxidation and the perchlorate reduction reactions, thus minimizing issues related to secondary water quality due to residual substrate and other fermentation products [49].

Although perchlorate reduction in microbial fuel cells still requires further study, data and findings from previous experimental investigations [27,28,48,49,50] were used to design the model set up for this case study. The reaction network studied is illustrated in Figure 6b. In the cathode, perchlorate reduction to chlorine occurs in four reactions in series [28]. Electrons for these reductive processes are supplied from the acetate oxidized in the anode. Acetoclastic methanogenesis is also accounted for in the anode chamber.

## 5. Conclusions

A developed model based on first principles aimed at prediction of chemical speciation and microbial activities and including a consistent electrical model describing current-to-potential interactions, can be applied to evaluate a wide range of MET processes in terms of their expected chemical and microbiological behavior. This constitutes a valuable tool to inform design, configuration, and control strategies. The model developed captures complex interactions and appears capable of predicting expected chemistry and electrical phenomena in multi-substrate, multi-population microbial MET systems at different configurations. These include power-producing MFC or power supplied MEC with possibly multiple electrode reactions either in parallel or in series. The model simulations showed responses to changes in input variables (applied voltage, membrane type, influent flow rates and concentrations), which are consistent with the expected behavior of the MET case studies presented and therefore bring insights into overall process dynamic interactions.

## Supplementary Materials

Supplementary materials can be found at www.mdpi.com/1422-0067/18/1/86/s1.
