# Supplementary material for: A Multiple Reaction Modelling Framework for Microbial Electrochemical Technologies
Source: Int J Mol Sci. 2017 Jan 4;18(1):86. doi: 10.3390/ijms18010086 (PMC5297720; doi:10.3390/ijms18010086)
Supplement: Supplementary file 1 [file ijms-18-00086-s001.pdf]

Supplementary Materials: A Multiple Reaction Modelling Framework for Microbial Electrochemical Technologies

Tolutola Oyetunde, Priyangshu M. Sharma, Farrukh Ahmad and Jorge Rodríguez

Stoichiometry of microbial growth

Acetoclastic methanogenesis

$$CH_3COO^- + 0.019NH_4{}^+ + 0.98\,H^+ \rightarrow 0.95\,CH_4 + 0.92CO_2 + 0.04H_2O + 0.095X$$

Acetate oxidation by electroactive bacteria

$$CH_3COO^- + 0.02NH_4{}^+ + 1.85H_2O \rightarrow 1.9CO_2 + 6.6H^+ + 0.1X + 7.58e^-$$

Kinetic rate equations

Rate of acetate consumption by acetoclastic methanogens

$$rS_{Acm} = q_{ac}^{max} \cdot \frac{S_{ac}}{K_s + S_{ac}} \cdot \frac{S_{in}}{K_{in} + S_{in}} X_{ac} \cdot I_{ph} \cdot I_{NH_3}$$

Rate of electroactive acetate oxidation(nernst-monod equation)

$$rS_{Acox} = q_{ac\_e}^{max} \cdot \frac{S_{ac}}{K_{se} + S_{ac}} \cdot \frac{S_{in}}{K_{in} + S_{in}} X_{ac\_e} \cdot I_{ph} \cdot \frac{\eta_{ac}^{act}}{KsE + \eta_{ac}^{act}}$$

Rate of transport between liquid and gaseous phases

$$rT_{CO2} = kLa. \left(S_{CO_{2(aq)}} - S_{CO_{2(g)}}/H\right)$$

$$CO_{2(g)} \rightleftharpoons CO_{2(aq)}$$

$$CO_{2(aq)} + H_2O \rightleftharpoons H_2CO_3 \rightleftharpoons H^+ + HCO_3{}^- \rightleftharpoons 2H^+ + CO_3{}^{2-}$$

Figure S1. Example microbial stoichiometric equations and rates.

$$\mathit{Cell\ Voltage} = \mathit{Cathode\ bulk\ potential} - \mathit{Anode\ bulk\ potential} - \mathit{Losses} + \mathit{applied\ voltage}$$

Activation losses of the anode reaction

(Ohmic) Losses caused by internal resistance

$$V_{cell} = E^{\emptyset}{}_{CA} - E^{\emptyset}{}_{AN} - \eta_{AN}^{cp} - \eta_{AN}^{act} - \eta_{CA}^{cp} - \eta_{CA}^{act} - I.Rint + V_{applied}$$

Maximum theoretical

Anode concentration polarization losses

Cathode concentration polarization losses

Activation losses of the cathode reaction

$$V_{cell} = \underbrace{\left(E^{\emptyset}{}_{CA} - \eta_{CA}^{cp} - \eta_{CA}^{act}\right)} - \underbrace{\left(E^{\emptyset}{}_{AN} + \eta_{AN}^{cp} + \eta_{AN}^{act}\right)} - I.R_{int} + V_{applied}$$

$$V_{cell} = E_{CA} - E_{AN} - I.R_{int} + V_{applied}$$

Activation losses (electrode kinetic limitations)

$$I_{AN\_max} = q_{ac\_e}^{max} \cdot \frac{S_{ac}}{K_{se} + S_{ac}} \cdot \frac{S_{in}}{K_{in} + S_{in}} X_{ac\_e} . I_{ph} . (F.V_{AN} \cdot y_e)$$

$$\eta_{AN}^{act} = \frac{KsE.I_{AN}}{\left(I_{AN\_max} - I_{AN}\right)}$$

Nernst equation (Gibbs free energy and potential)

$$E^{\emptyset}{}_{AN} = E^{\emptyset}{}_{AN\_ref} + j\frac{RT}{v_e F}\ln\prod_i a_i^{v_i}$$

$$E^{\emptyset}{}_{AN\_ref} = {\Delta Gref^{\emptyset}}\Big/{-jv_e F}$$

Concentration losses (diffusion limitations

$$I_{AN\_diff} = (F.V_{AN} \cdot y_e) \cdot \frac{D_{ac}}{d_{AN}} . S_{ac} . A_{AN}$$

$$\eta_{AN}^{cp} = E^{\emptyset'}{}_{AN} - E^{\emptyset}{}_{AN}$$

$$E^{\emptyset'}{}_{AN} = f(S'_i)$$

$$S'_i = S_i \cdot \left(1 + j * \left(\frac{I_{AN}}{I_{AN\_diff}}\right)\right)$$

Figure S2. Electrical model equations.

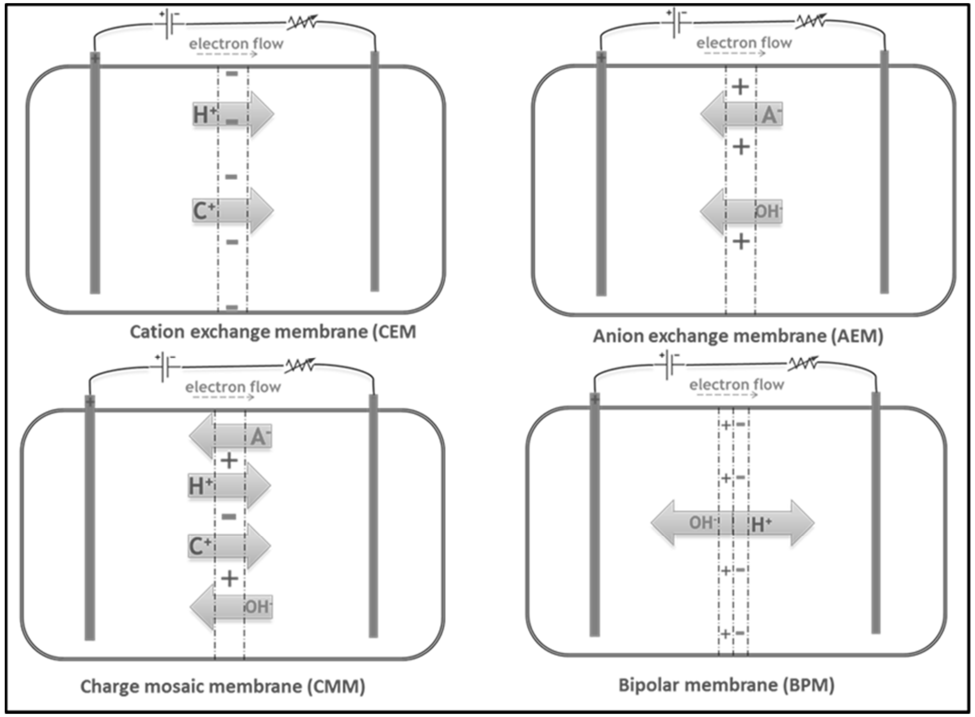

$$\frac{I_{AN}}{V_{AN}F} = \sum_i J_i \quad \Rightarrow \quad J_i = t_i.\frac{I_{AN}}{V_{AN}F}, \qquad \sum_i t_i = 1$$

$$t_i = \frac{z_i.R_i.\sigma_i}{\sum_i z_i.R_i.\sigma_i} \qquad \sigma_i = \lambda_i.Ci$$

Figure S3. Modeling ionic flow across membrane.

Table S1. Model parameters used in the ethanol/butanol and perchlorate remediation case studies.

Parameter	Symbol	Value	Units	Comments
Max. oxidative acetate consumption rate	qSaceAN_max	1.04×10^{-4}	molSac/molXace·s	assumed(3 mole- per mole substrate per hour)
Max. oxidative hyrogen consumption rate	qSh2eAN_max	4.17×10^{-4}	molSh2/molXh2e·s	assumed(3 mole- per mole substrate per hour)
Max. reductive acetate consumption rate	qSaceCA_max	2.08×10^{-4}	molSac/molXace·s	assumed(3 mole- per mole substrate per hour)
Max. reductive butyrate consumption rate	qSbueCA_max	2.08×10^{-4}	molSbu/molXbue·s	assumed(3 mole- per mole substrate per hour)
Max. reductive proton consumption rate	qSh2eCA_max	8.33×10^{-4}	molSh+/molXh2e·s	assumed(3 mole- per mole substrate per hour)
Max. reductive ClO_4^- consumption rate	qSclo4_max	1.150×10^{-4}	molSclox/molXeaa·s	assumed
Max. reductive ClO_3^- consumption rate	qSclo3_max	1.150×10^{-4}	molSclox/molXeaa·s	assumed
Max. reductive ClO_2^- consumption rate	qSclo2_max	1.150×10^{-4}	molSclox/molXeaa·s	assumed
Max. reductive ClO^- consumption rate	qSclo_max	1.150×10^{-4}	molSclox/molXeaa·s	assumed
Max acetate consumption rate	qSacm_max	4.86×10^{-5}	molSac/molXacm·s	ADM1
Max butyrate consumption rate	qSbu_max	4.86×10^{-5}	molSbu/molCxc4·s	ADM1
Max hydrogen consumption rate	qSh2_max	8.51×10^{-4}	molSh2/molCxh2·s	ADM1
Monod half saturation constant(acetate)	Ks_ac	2.34×10^{-3}	molSac/L	ADM1
Monod half saturation constant (inorganic nitrogen)	Ks_in	1.00×10^{-4}	molSin/L	ADM1
Monod half saturation constant (butyrate)	Ks_bu	1.88×10^{-3}	molSbu/L	ADM1
Monod half saturation constant (hydrogen)	Ks_h2	1.56×10^{-6}	molSh2/L	ADM1
Monod electroactive half saturation constant (acetate)	Ks_ace	1.91×10^{-3}	molSac/L	[38]
Monod electroactive half saturation constant (butyrate)	Ks_bue	8.55×10^{-4}	molSbu/L	[38]
Monod electroactive half saturation constant (hydrogen)	Ks_h2e	1.25×10^{-6}	molSh2/L	assumed(20% less than anaerobic fermentation)
Monod electroactive half saturation constant (perchlorate)	Ks_clox	0.000779095	molClOx/L	
pH upper limit	pHul	5	[]	ADM1
pH lower limit	pHll	4	[]	ADM1
pH upper limit (acetoclastic methanogens)	pHll_ac	6	[]	ADM1
pH lower limit (acetoclastic methanogens)	pHul_ac	7	[]	ADM1
Ammonia inhibition constant	Ki_nh3_ac	1.80×10^{-3}	molSnh3/L	ADM1
Decay rate constant	kd	2.31×10^{-7}	1/s	ADM1
Acetate diffusion coefficient	Dac	1.21×10^{-9}	m^2/s	http://www.biofilmbook.com
Hydrogen diffusion coefficient	Dh2	4.50×10^{-9}	m^2/s	http://www.biofilmbook.com
Butyrate diffusion coefficient	Dbut	8.70×10^{-10}	m^2/s	http://www.biofilmbook.com
Perchlorate diffusion coefficient	Dclox	1.00×10^{-9}	m^2/s	

Table S1. *Cont.*

Parameter	Symbol	Value	Units	Comments
Characteristic anode length	dAN	1.00×10^{-4}	m	assumed(depends on geometry)
Characteristic cathode length	dCA	1.00×10^{-4}	m	assumed(depends on geometry)
Gas- liquid transfer coefficient	kLa	2.31×10^{-5}	1/s	ADM1(a measure of mixing)
Electrical monod term (acetate at anode)	KsE_ac_an	1.00×10^{-4}	V	assumed
Electrical monod term (butyrate at anode)	KsE_bu_an	1.00×10^{-4}	V	assumed
Electrical monod term (hydrogen at anode)	KsE_h2_an	1.00×10^{-4}	V	assumed
Electrical monod term (acetate at cathode)	KsE_ac_ca	1.00×10^{-4}	V	assumed
Electrical monod term (butyrate at cathode)	KsE_bu_ca	1.00×10^{-4}	V	assumed
Electrical monod term (hydrogen at cathode)	KsE_h2_ca	1.00×10^{-4}	V	assumed
Electrical monod term (ClO_4^- at cathode)	KsE_clo4_ca	0.0005	V	assumed
Electrical monod term (ClO_3^- at cathode)	KsE_clo3_ca	0.0005	V	assumed
Electrical monod term (ClO_2^- at cathode)	KsE_clo2_ca	0.0005	V	assumed
Electrical monod term (ClO^- at cathode)	KsE_clo_ca	0.0005	V	assumed
Yield(electroactive acetate oxidation)	Y_ac_an_e	0.100	molX/molAc	assumed(5% more than anaerobic fermentation)
Yield(electroactive hydrogen oxidation)	Y_h2_an_e	0.030	molX/molH2	assumed(5% more than anaerobic fermentation)
Yield(acetoclastic Methanogenesis)	Y_acm	0.095	molX/molAc	ADM1
Yield(hydrotrophic Methanogenesis)	Y_h2m	0.029	molX/molH2	ADM1
Yield(Butyric reduction to acetate)	Y_but_ac	0.286	molX/molBu	ADM1
Yield(electroactive Butyrate reduction)	Y_but_ca_e	0.285	molX/molBu	assumed(0.25% less than anaerobic fermentation)
Yield(butyrate reduction by hydrogen)	Y_but_h2	0.286	molX/molBu	assumed(adm1)
Yield(acetate reduction by hydrogen)	Y_ac_h2	0.095	molX/molAc	assumed(adm1)
Yield(electroactive proton reduction)	Y_h+_e	0.029	molX/molH+	assumed(0.25% less than anaerobic fermentation)
Yield(electroactive acetate reduction)	Y_ac_ca_e	0.095	molX/molAc	assumed(0.25% less than anaerobic fermentation)
Yield(electroactive ClO_4^- reduction)	Ye_clo4	2.559	mole/molClO_4	assumed
Yield(electroactive ClO_3^- reduction)	Ye_clo3	2.503	mole/molClO_3	assumed
Yield(electroactive ClO_2^- reduction)	Ye_clo2	2.777	mole/molClO_2	assumed
Yield(electroactive ClO^- reduction)	Ye_clo	2.925	mole/molClO	assumed
Maximum biomass concentration	Xtmax	0.132	kmol/m^3 (M)	[17]

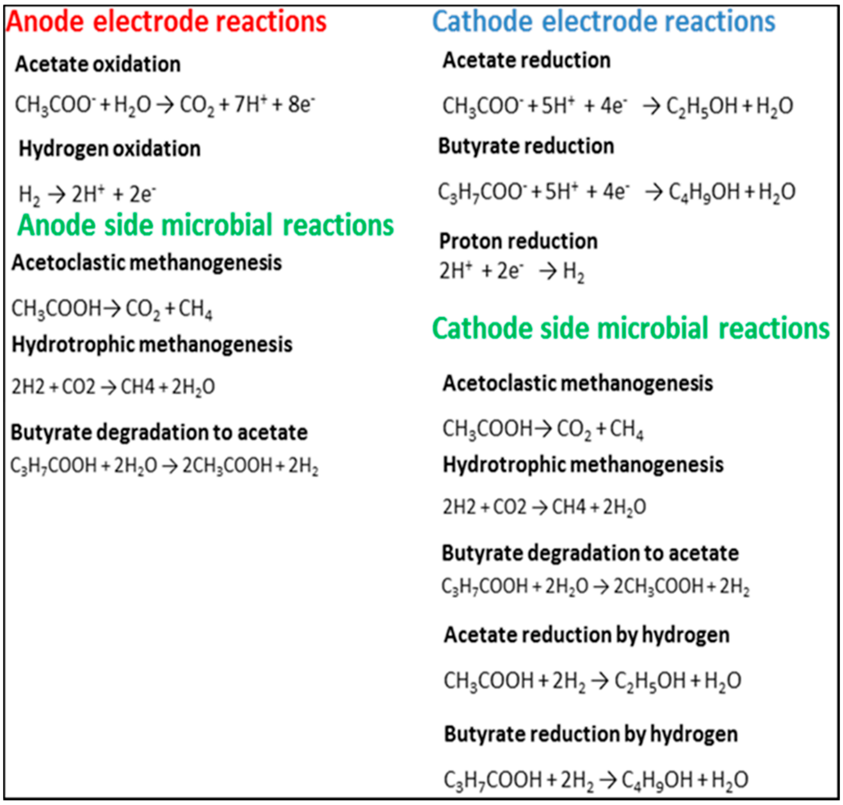

Figure S4. Electrode reactions for the ethanol/butanol case study.

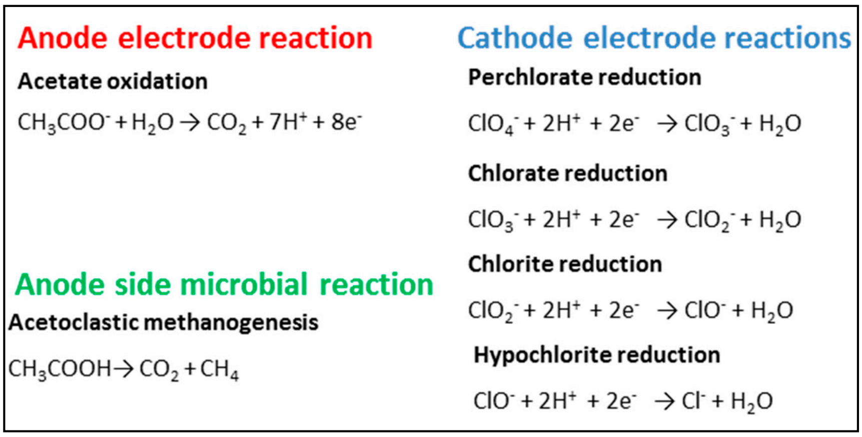

Figure S5. Electrode reactions for the perchlorate remediation case study.
